# PIEZO2 and perineal mechanosensation are essential for sexual function

**DOI:** 10.1126/science.adg0144

**Published:** 2023-08-24

**Authors:** Ruby M. Lam, Lars J. von Buchholtz, Melanie Falgairolle, Jennifer Osborne, Eleni Frangos, M. Rocio Servin-Vences, Maximilian Nagel, Minh Q. Nguyen, Monessha Jayabalan, Dimah Saade, Ardem Patapoutian, Carsten G. Bönnemann, Nicholas J. P. Ryba, Alexander T. Chesler

**Affiliations:** 1 National Center for Complementary and Integrative Health (NCCIH), Bethesda, MD 20892, USA.; 2 Brown-National Institutes of Health Graduate Partnerships Program, Brown University, Providence, RI 02912, USA.; 3 National Institute of Dental and Craniofacial Research, Bethesda, MD 20892, USA.; 4 Howard Hughes Medical Institute, Department of Neuroscience, Dorris Neuroscience Center, The Scripps Research Institute, La Jolla, CA 92037, USA.; 5 National Institute of Neurological Disorders and Stroke, Bethesda, MD 20892, USA.

## Abstract

Despite the potential importance of genital mechanosensation for sexual reproduction, little is known about how perineal touch influences mating. We explored how mechanosensation affords exquisite awareness of the genitals and controls reproduction in mice and humans. Using genetic strategies and in vivo functional imaging, we demonstrated that the mechanosensitive ion channel PIEZO2 (piezo-type mechanosensitive ion channel component 2) is necessary for behavioral sensitivity to perineal touch. PIEZO2 function is needed for triggering a touch-evoked erection reflex and successful mating in both male and female mice. Humans with complete loss of PIEZO2 function have genital hyposensitivity and experience no direct pleasure from gentle touch or vibration. Together, our results help explain how perineal mechanoreceptors detect the gentlest of stimuli and trigger physiologically important sexual responses, thus providing a platform for exploring the sensory basis of sexual pleasure and its relationship to affective touch.

Sexual reproduction is a fundamental driver for animal behavior, and adaptations required for courtship, including sexual ornamentation and ritual displays, are cornerstones of evolutionary theory (1–3). Visual, auditory, and olfactory cues promote mating in various mammalian species (4–8); however, the act of copulation itself can be considered a specialized form of touch endowed with its own cortical field (9). Although the discovery of the mechanically gated ion-channel PIEZO2 (piezo-type mechanosensitive ion channel component 2) (10) has spurred remarkable progress in our understanding of discriminative touch (11, 12), far less is known about mechanosensation in the genitals (13, 14), including how it triggers physiological responses and elicits pleasure. We hypothesized that sexual touch might exhibit unusual response specialization to control mating and provide affective and motivational feedback. We also expected that there would be sexual dimorphism both in sensation and in responses triggered by genital-innervating mechanosensors. To test these hypotheses, we developed a series of behavioral and functional imaging assays to probe the role of PIEZO2 in genital mechanosensation and sexual function. In addition, by exploring the impact of PIEZO2 loss of function caused by a rare inherited syndrome, we determined how these findings relate to human sexual experience.

## Unusual sensitivity and PIEZO2 dependence of perineal touch

**Fig. 1.**
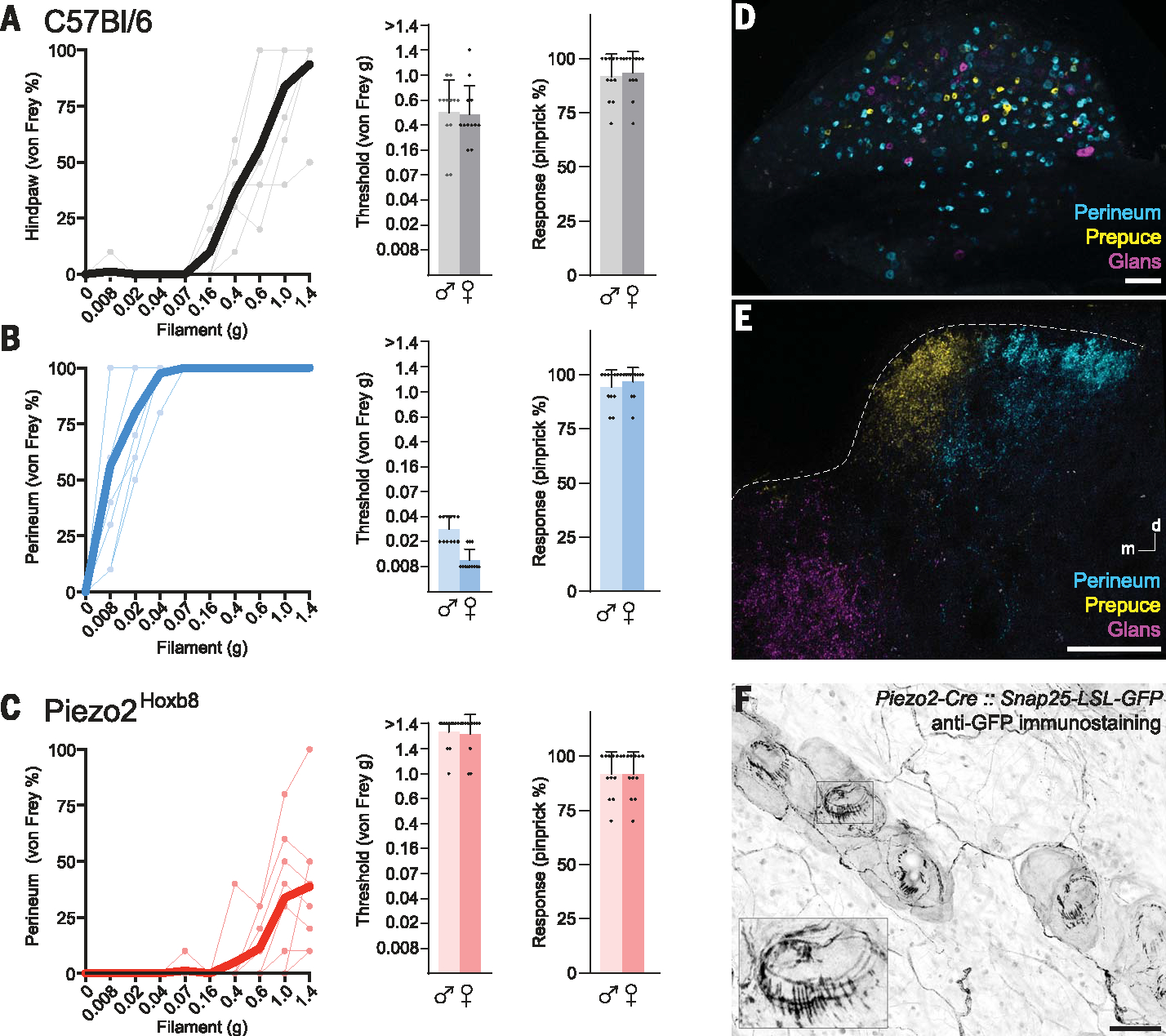
Behavioral sensitivity of mice to perineal touch and underlying anatomy. (**A** to **C**) Reaction of mice to punctate touch (A) wild-type hind-paw, (B) wild-type perineum, and (C) Piezo2^Hoxb8^ perineum. (Left) Example responses for individual mice (points and thin lines; four males and four females) and mean (solid lines) to a series of calibrated von Frey filaments (grams, each tested 10 times per mouse). (Middle) Quantitation of von Frey threshold (≥5/10; *n* = 12 males and 12 females). Thresholds are different between all three groups [one-way analysis of variance (ANOVA) on ranks *P* < 0.001]. Wild-type females exhibited a lower perineal touch threshold than that of males (Mann-Whitney *t* test; *P* < 0.0001); there were no significant differences in other responses (supplementary materials, statistical reporting). (**D** and **E**) Triple-color retrograde CTB tracing from the perineum (cyan), prepuce (yellow), and glans (magenta) showing (D) cell bodies of lumbar-sacral sensory neurons in the DRG and (E) termini in the dorsal spinal cord. The dotted line indicates approximate extent of dorsal horn. In (E) and (F), *n* = 4 mice. Scale bars, 100 μm. (**F**) Anatomy of sensory ending of *Piezo2*-expressing sensory neurons in the perineum. (Inset) A magnified view of a single hair (boxed) highlighting prominent lanceolate and circumferential endings (*n* = 2 males and 1 female). Scale bar, 50 μm.

A standard touch sensitivity test uses calibrated von Frey filaments to measure detection threshold. In mice, von Frey sensitivity of the glabrous hind-paw and hairy skin of the face have similar withdrawal thresholds (15–17) despite very different patterns of innervation (18). We adapted this assay to compare stimulation of the perineum (the region extending from the anus to the genitals in male and female mice) with that of the plantar surface of the paw. In the hind-paw assay, mice respond by withdrawing the paw with no indication of pain or distress. Our data (Fig. 1A) match literature reports, with filaments ≥0.4 g eliciting responses in the majority of trials, but filaments ≤0.16 g rarely provoking reaction (15, 17). By contrast, stimulation of the perineum evoked a highly stereotyped startle and investigative response (movie S1) both in male and female mice. Even the finest filament available (0.008 g) elicited this reaction from every animal (Fig. 1B), demonstrating exquisite sensitivity of the perineum to forces below those that reliably trigger responses from other sites, even in mice with profound allodynia (15, 17); female mice were marginally but consistently more sensitive than males (Fig. 1B).

The mechanically activated ion channel PIEZO2 is essential for discriminative touch in mice and humans (11, 12). We anticipated that this mechanoreceptor would also be responsible for the sensitivity of the perineum. *Piezo2*-null mice die as neonates (19); therefore, we generated conditional genetic deletions using a *Hoxb8-Cre* line (Piezo2^Hoxb8^) to target cells below the mid-thoracic region (17). We used this strategy to assess the role of PIEZO2 in perigenital sensation and observed profound loss of behavioral response to von Frey filaments (Fig. 1C), with the highest force tested (1.4 g) eliciting responses in only ~40% of trials (movie S1). Local inhibition of the perineum with lidocaine attenuated von Frey responses of controls (fig. S1), and Piezo2^Hoxb8^ responses to noxious mechanical pinprick were indistinguishable from those of controls (Fig. 1C and movie S1). Therefore, the Piezo2^Hoxb8^ deficit is likely to be sensory rather than related to a movement disorder (20). These experiments demonstrated that PIEZO2 is crucial for triggering behavioral responses to the gentlest of perigenital touch in mice; without this touch receptor, von Frey stimulation of the genital region rarely elicited responses even at intensities considered noxious.

We previously studied a rare cohort of people with biallelic loss-of-function variants of *PIEZO2* who have sensory deficits fully consistent with those described in animal models (11, 21). In our clinical interviews, five adult human subjects with PIEZO2-deficiency syndrome (three male and two female) reported severe hyposensitivity in genital sensation (table S1); however, comprehensive quantitative testing has not been possible. One individual adult male consented to quantitative sensory testing of his genitalia during clinical evaluation. His penile von Frey detection threshold (3.1 ± 1.5 g) was far higher than values reported in the literature: 0.3 to 0.6 g in a similar location (22). He had difficulty detecting pressure below 1 kg/cm^2^ at the midshaft and was insensitive to strong vibration at 50 and 100 Hz, which is consistent with our findings in mice. By contrast, literature values for penile fine-touch pressure thresholds in a range of healthy men are far lower (23), and vibration is normally readily detected (23).

## Anatomy of perineal neurons

Somatosensory neurons in the lower body have soma in lumbar (L1 to L6) and sacral (S1 to S4) dorsal root ganglia (DRG) (24). However, few details about the types or sensitivity of neurons that target the genitals are known. Multicolor cholera toxin subunit-b (CTB) tracing from both hind-paw and genitals robustly labeled neurons in S1 and S2 DRG (fig. S2A) and distinguished neurons that target perigenital subregions (Fig. 1, D and E). Injections to the perineum, prepuce, and glans (male mice) or vaginal opening (female mice) resulted in largely nonoverlapping labeling of neurons with a range of cell diameters (Fig. 1D and fig. S2B). In both sexes, dense projections targeted the (L6 to S2) spinal cord, with perineal neurons (Fig. 1, D and E, cyan) synapsing in the touch recipient zone (25) of the lateral dorsal horn (Fig. 1E and fig. S2C). Neurons innervating male prepuce (Fig. 1, D and E, yellow) projected to a medial portion of the touch zone (Fig. 1E), whereas glans axons (Fig. 1, D and E, magenta) terminated proximal to the central canal (Fig. 1E). In females, axons from the vaginal opening targeted the medial dorsal horn, whereas those from the prepuce, which includes the clitoris, closely resembled those from the glans in males.

To visualize the peripheral anatomy of touch neurons in the perineum, we generated mice in which *Piezo2*-expressing neurons were selectively labeled by crossing a *Piezo2-Cre* allele (26) into a neural-specific *Snap25-LSL-GFP* reporter line (27). Green fluorescent protein (GFP) staining of cleared skin demonstrated that the perineum was densely innervated with lanceolate and circumferential endings surrounding hair follicles (Fig. 1F), which is consistent with innervation by a broad range of low-threshold mechanosensory neurons (LTMRs) and the PIEZO*2*-dependent behavioral sensitivity of mice to perineal touch.

## Perineal sensory neurons exhibit high sensitivity to punctate stimulation

**Fig. 2.**
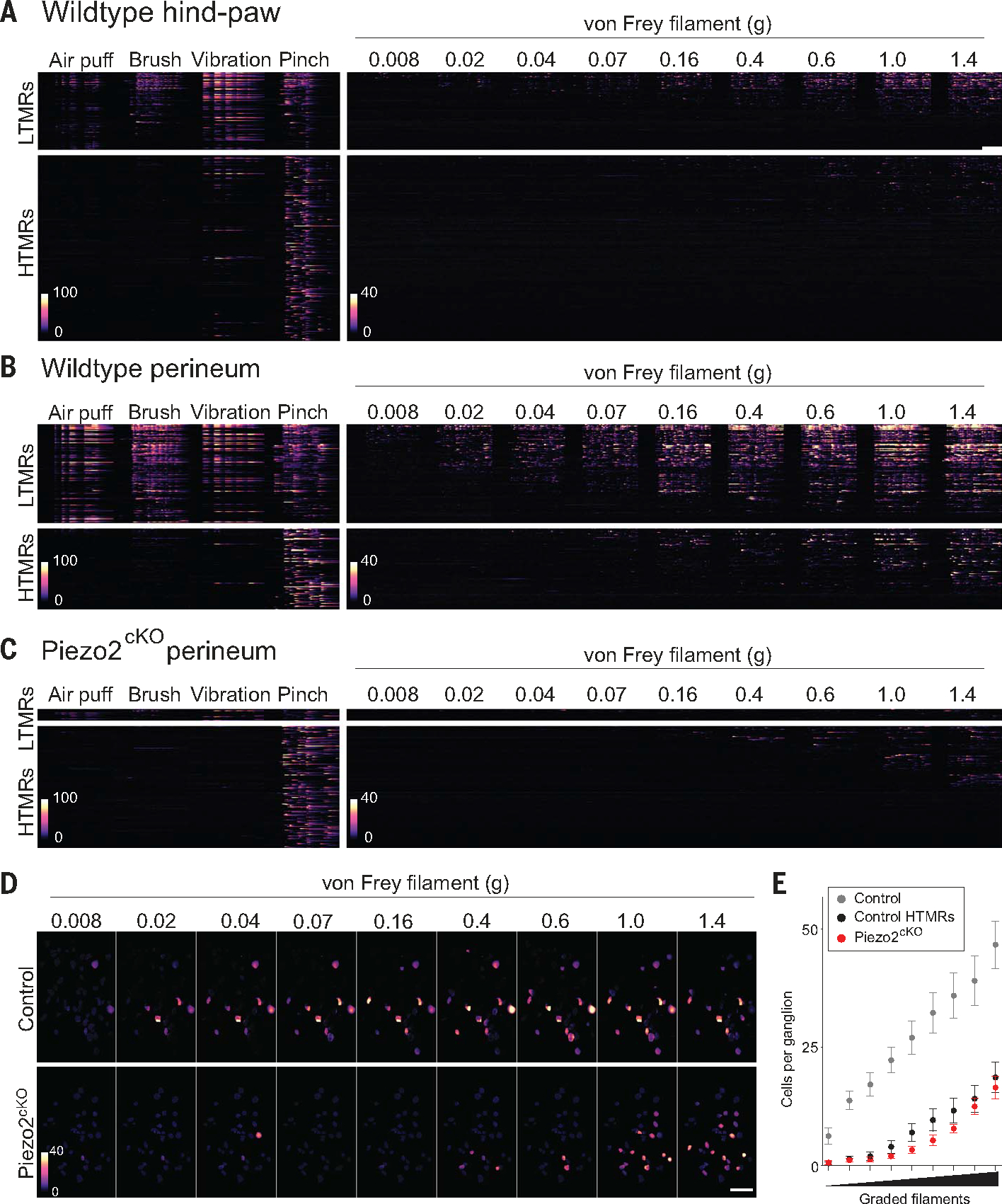
Functional characterization of perineal mechanoreceptors and role of PIEZO2. (**A** to **C**) Heatmaps representing calcium (GCaMP6f) responses to (left) repetitive application of naturalistic stimuli and (right) graded von Frey stimulation. LTMRs and HTMRs are separated, and relative fluorescence changes (D*F*/*F*) are colored as indicated. Scale bar, 10 s. (A) Wild-type hind-paw, *n* = 4 mice. (B) Wild-type perineum, *n* = 4 mice. (C) Piezo2^cKO^ perineum, *n* = 6 mice. Additional analysis is provided in figs. S3 and S4. (**D**) Spatial activity maps of control and Piezo2^cKO^ neurons to von Frey filaments. Scale indicates response intensity. Scale bar, 100 μm. (**E**) Quantitation of von Frey responsive neurons in control mice (gray), Piezo2^cKO^ mice (red), and response profile of control HTMRs (black) (mean ± SEM, *n* = 8 control mice, *n* = 6 Piezo2^cKO^ mice). Piezo2^cKO^ mice had fewer von Frey responsive neurons at all filament strengths (Mann Whitney *U* test; *P* < 0.0087).

We developed a sacral ganglia imaging preparation to compare neural responses to a range of gentle and intense mechanical stimuli (28) applied to the hind-paw and perineum (fig. S3 and movie S2). Neurons innervating paw glabrous skin divided into LTMRs and high-threshold mechanosensory neurons (HTMRs) on the basis of their response selectivity (Fig. 2A; fig. S3; and supplementary materials, materials and methods). HTMRs that innervate the paw outnumbered LTMRs by a factor of 2. In particular, LTMRs exhibited graded von Frey sensitivity (Fig. 2A and fig. S3D) and could be activated by forces as low as 0.008 g, whereas HTMRs were essentially silent at forces below 0.4 g, matching behavioral withdrawal threshold and implicating HTMRs in this response. By contrast, perineal sensation was dominated by LTMRs, with ~60% of mechanosensory neurons responding to gentle stimuli (Fig. 2B and figs. S3 and S4) and broad similarity between male and female mice (fig. S4). Almost all perineal mechanosensors could be activated by von Frey stimulation (Fig. 2B, figs. S3, S4), and their calcium (GCaMP) signals were markedly stronger than for paw-innervating neurons (fig. S3D). Few HTMRs responded to the fine filaments that reliably evoked behavioral responses (Fig. 1B and movie S1). Therefore, both male and female mice are attuned to perineal LTMR input, and the stereotyped reaction to genital touch is not a sign of pain.

## A broad role for PIEZO2 in perineal sensation

To measure the contribution of PIEZO2 to perigenital touch and to dissect the mechanism underlying the extreme sensitivity to von Frey stimulation, we next used the sacral imaging platform to selectively image cells that lack this stretch-gated ion channel (fig. S4D). As expected, deletion of *Piezo2* (Piezo2^cKO^) dramatically affected the mechanosensitivity of genital-innervating neurons. The great majority of responses to air puff, vibration, and brush were eliminated. Thus, mechanosensory neurons were only stimulated by pinch and were almost exclusively HTMRs (fig. S4E). The overall number of HTMRs was similar between wild-type and Piezo2^cKO^ mice (fig. S4F), which is consistent with earlier studies (17, 20, 21). Piezo2^cKO^ mice responses to von Frey stimulation were substantially reduced and recapitulated those of control perineal HTMRs (Fig. 2, C to E). These results likely explain the absence of behavioral reactions to von Frey stimulation in Piezo2^Hoxb8^ mice (Fig. 1C), support the hypothesis that perineal LTMRs drive this characteristic withdrawal in wild-type mice (movie S1), and are consistent with human reports and sensory testing (table S1).

## A subset of touch neurons is required for mechanically induced erection responses

**Fig. 3.**
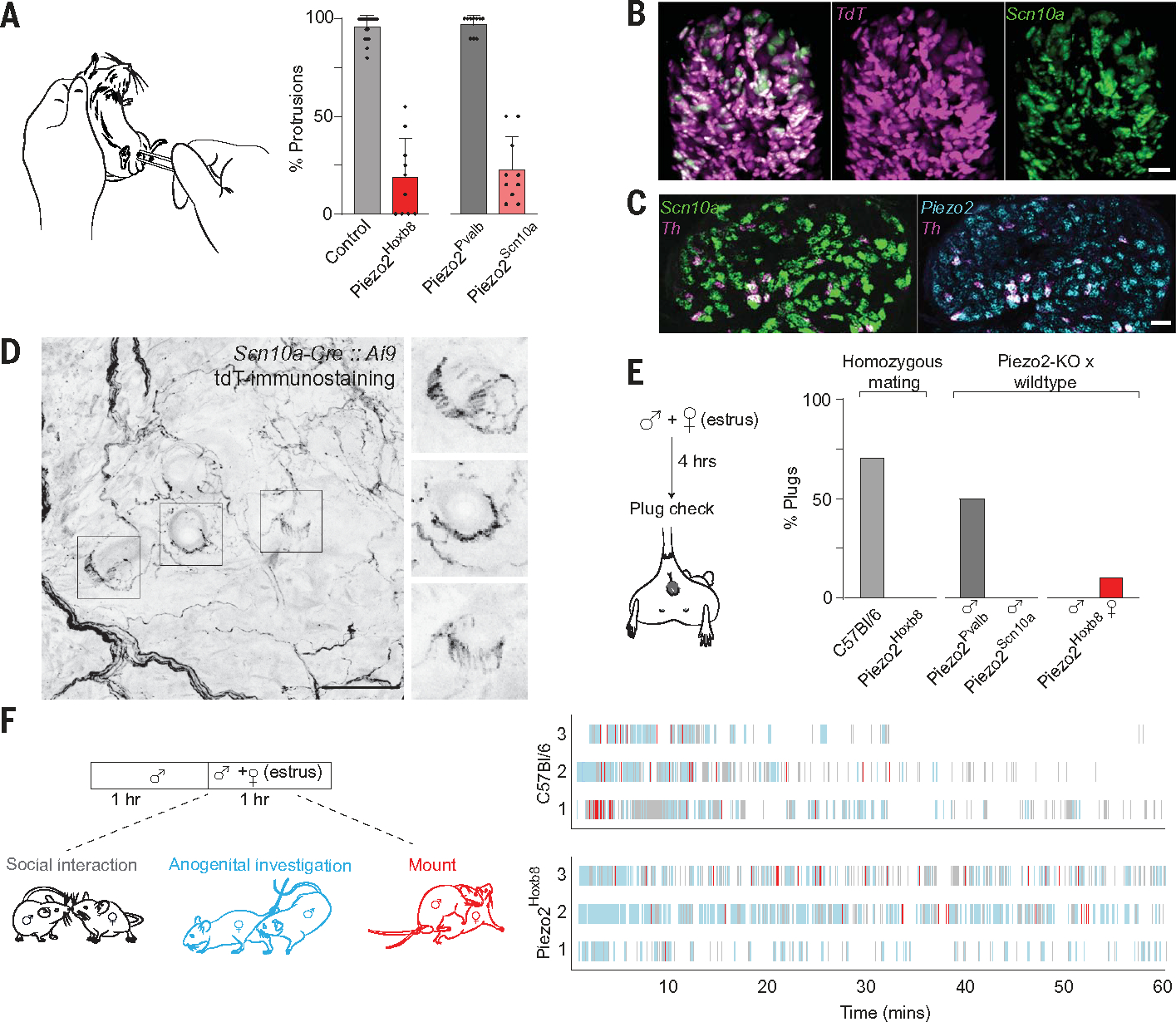
A role for PIEZO2-dependent perineal mechanosensation in mating. (**A**) Physiological responses of male mice to perineal stimulation with transparent soft tubing. Penile protrusion was scored for two sets of 10 trials. Bars indicate mean ± SEM, and points indicate individual responses. Control versus Piezo2^Hoxb8^ mice and Piezo2^Pvalb^ versus Piezo2^Scn10a^ mice were different (Mann-Whitney *U* test; *P* < 0.0001; *n* = 18 control mice; *n* = 10 *Piezo2*-deleted mice). (**B**) Representative whole-mount ISH of sacral ganglion showing faithful recombination [*tdTomato* (*TdT*); magenta] of *Scn10a-Cre* mouse in *Scn10a* (green) neurons; >90% (670 of 738) *TdT* cells expressed *Scn10a* (*n* = 3 ganglia). Scale bar, 50 μm. (**C**) Example ISH of sacral ganglion section probed for *Th* (magenta), *Scn10a* (green), and *Piezo2* (cyan), illustrating expression of *Scn10a* and *Piezo2* in cLTMRs identified with *Th* (*n* = 6 ganglia). Scale bar, 50 μm. (**D**) Anatomy of sensory ending of *Scn10a*-expressing sensory neurons in the perineum (maximum projection, full-stack). (Right) Magnified and focal views of single hairs (boxed at left), highlighting lanceolate endings of *Scn10a-Cre*–labeled neurons (*n* = 2 male mice). (**E**) Successful mating scored by vaginal plugs (*n* = 10 mice). Differences are significant for C57Bl/6 versus Piezo2^Hoxb8^ mice (*P* = 0.0031) and Piezo2^Pvalb^ versus Piezo2^Scn10a^ mice (*P* = 0.0325) (Fisher’s exact test, two-tailed). (Right) Mating success for female and male Piezo2^Hoxb8^ mice with C57Bl/6 partners. (**F**) Representative ethogram plots showing sexual motivation of three isogenic pairings of C57Bl/6 and 3 Piezo2^Hoxb8^ mice: social interaction (gray), anogenital investigation (pale blue), and mounting attempts (red).

Perineal investigation and touch precedes mating in many species, including mice (29). These behaviors are linked to motivational drive in both partners and trigger physiological reflexes. For example, gentle retraction of the prepuce induces penile cupping (erection) and flipping (ejaculation) in spinalized rodents (30). We reasoned that mechanosensory input drives the erection reflex and developed an assay to monitor this in restrained awake mice. A soft, transparent tube was used to gently retract the prepuce, allowing the physiological erection reflex (extension of the penis into the tube) to be scored. Wild-type controls responded in almost every single trial (Fig. 3A); isoflurane anesthesia completely eliminated responses, and local numbing of the perineum with lidocaine greatly dampened the reflex (fig. S5). As we anticipated, Piezo2^Hoxb8^ mice only very rarely exhibited penile extension in response to prepuce retraction (Fig. 3A).

Piezo2^Hoxb8^ mice exhibit broad loss of touch but also have proprioceptive (and potentially other mechanosensory) deficits (17, 28). Therefore, we examined mice with more selective *Piezo2* deletions. Piezo2^Pvalb^ mice (in which *Piezo2* is inactivated by using *parvalbumin*-driven *Cre*) lack proprioceptive input but still respond to gentle touch (20). These mice had perfectly normal responses to prepuce retraction (Fig. 3A) despite severe ataxia. We also generated *Piezo2* deletions using an *Scn10a-Cre* line, which is commonly used to target a broad range of nociceptors, including HTMRs (31). Perineal HTMR responses are PIEZO2 independent (Fig. 2 and fig. S4); therefore, these mice (Piezo2^Scn10a^) were predicted to have normal proprioception, touch, and consequently erection reflexes. Piezo2^Scn10a^ mice walked with normal gait, and recombination of *Scn10a-Cre* in sacral ganglia neurons was faithful (Fig. 3, B and C, and fig. S6A), with only a few large-diameter *Scn10a*-negative LTMRs labeled (fig. S6A). Nonetheless, Piezo2^Scn10a^ mice displayed severe deficits in their erection reflex, closely recapitulating the phenotype of Piezo2^Hoxb8^ animals (Fig. 3A) and the effects of lidocaine (fig. S5A). Single-cell sequencing data from lumbar DRG (32) and trigeminal neurons (33) validate *Scn10a* as a robust marker for nociceptors but reveal expression in c-fiber LTMRs (cLTMRs). We used in situ hybridization (ISH) to confirm coexpression of *Scn10a*, the cLTMR marker *Tyrosine hydroxylase* (*Th*) (24), and *Piezo2* in sacral ganglia (Fig. 3C), with only very limited recombination in other potential LTMRs (fig. S6A). Because cLTMR responses to gentle mechanical stimulation depend on *Piezo2* expression (34), these data strongly suggest a causal role for perineal cLTMR input in triggering the erection reflex. Consistent with this hypothesis, tdTomato–positive lanceolate endings (typical of cLTMRs) surround perineal hair follicles in *Scn10a-Cre*, *Ai9* mice (Fig. 3D). Moreover, functional imaging of perineal touch responses in *Scn10a-Cre*, *Ai95* mice revealed that neurons responding to gentle mechanical stimuli (fig. S6, B and C) had uniform small diameters, as would be expected for cLTMRs (24, 34).

## Severely impaired sexual function in mice lacking PIEZO2

Loss of a touch-induced erection response in Piezo2^Hoxb8^ males should impair mating. Indeed, 10 pairs of mating-age Piezo2^Hoxb8^ males and females housed together for 6 months never produced pups, whereas wild-type (C57Bl/6) controls delivered 61 litters in this time (range, five to seven litters per pair). To assess copulatory success more directly, we also examined the frequency of vaginal plug formation after introducing virgin females in estrus to single housed males; to eliminate bias from prior experience, all mice were naïve. For C57Bl/6 mice, 7 from 10 homozygous pairings had plugs after 4 hours (Fig. 3E). By contrast, plugs were never seen for Piezo2^Hoxb8^ male mice when paired either with Piezo2^Hoxb8^ or wild-type females (Fig. 3E). As predicted from their normal erection reflexes, Piezo2^Pvalb^ males successfully mated with C57Bl/6 females despite severe ataxia (Fig. 3E). However, Piezo2^Scn10a^ males failed to plug receptive C57Bl/6 females, substantiating the importance of PIEZO2-dependent mechanosensory input for male mating behavior (Fig. 3E). Although loss of erection reflexes may explain why Piezo2^Hoxb8^ mice fail to breed, mechanosensation probably has additional roles in mating. For example, female mice have similar PIEZO2-dependent perineal mechanosensitivity to males (fig. S4) and are even more sensitive to perigenital touch (Fig. 1); Piezo2^Hoxb8^ females exhibited strong mating deficits when paired with wild-type males: 9 from 10 remained unplugged after 4 hours (Fig. 3E).

Ethogram analysis of female intruder assays (Fig. 3F) assess motivation by quantifying stereotyped male behaviors, including partner-grooming, anogenital chemosensory investigation, and mounting attempts (35, 36). We analyzed behavior for 1 hour after introduction of receptive females (supplementary materials, materials and methods). Control animals exhibited considerable variation in mating behavior (Fig. 3F) but in every case (*n* = 10 pairs of mice) engaged in chemosensory investigation and mounting attempts shortly after introduction of the female. Similarly, pairs of Piezo2^Hoxb8^ males and females (*n* = 10 pairs) (Fig. 3F) as well as male or female Piezo2^Hoxb8^ mice paired with C57Bl/6 partners (*n* = 10 pairs in each case) (fig. S5B) exhibited strong sexually motivated behavior, not very different from controls. However, Piezo2^Hoxb8^ males never achieved intromission, which was regularly observed in wild-type controls. Similarly, Piezo2^Scn10a^ males paired with receptive C57Bl/6 females showed normal sexual motivation (*n* = 10 males and 10 females) (fig. S5B) but without copulatory success (Fig. 3E). Moreover, Piezo2^Hoxb8^ females paired with C57Bl/6 males also engaged in premating behavior, including male mounting attempts (fig. S5B), but Piezo2^Hoxb8^ females adopted a sit-rejection posture, preventing intromission (37). These data show that mechanosensation plays a substantial role in productive mating and exposes dimorphic need for PIEZO2 and gentle touch in sexual function.

## Impact of PIEZO2 in human sexual experience

The genital sensation of a man with complete loss of PIEZO2 function and comprehensive touch- and proprioception-related studies of individuals with PIEZO2-deficiency syndrome (11, 21) demonstrate strongly conserved roles for PIEZO2 in mammalian mechanosensation. For humans, sexual experience is not simply related to reproduction but is central to large parts of many people’s social lives and behavior. Information from human clinical evaluations (*n* = 5; three men and two women) (table S1) provided several consistent themes about the role of gentle touch in sex. First, these individuals with biallelic loss of function (table S1A) had diagnostic clinical presentation, with loss of proprioception, absent vibration sensing, highly elevated touch threshold, and scoliosis but no cognitive difficulties, and all underwent puberty without clinically relevant problems. Second, all five people with PIEZO2 deficiency reported being sexually active and able to be aroused by physical genital stimulation, erotic thoughts, or videos, reflecting motivation seen in Piezo2^Hoxb8^ mice (Fig. 3F). Third, individuals with PIEZO2 deficiency reported delayed, attenuated, or absent physiological responses to gentle genital stimulation. This included clinical diagnosis of hypo-orgasmia for the male and anorgasmia for the female participants, which again is consistent with the animal model. However, the five people had strategies to compensate for deficits in genital sensation (table S1B).

## Discussion

Erogenous touch conveys different meanings according to circumstance; however, many key details remain unknown. We explored how deficits in PIEZO2-dependent mechanosensation interfere with perigenital sensation, physiological response, copulation, and reproduction. Our results demonstrate that PIEZO2-dependent touch is required for all of these in mice.

Anatomical studies have identified specialized corpuscles composed of myelinated afferents likely involved in genital sensation (38, 39). Our data strongly implicate an additional type of touch neuron, the perineal cLTMRs, as crucial drivers of sexual function. Previous studies in mice and humans suggest specialized roles for cLTMRs in conveying affective and pleasurable touch (40, 41). Thus, it is of note that five individuals without PIEZO2 function described sexual activity as satisfying and rewarding despite marked mechanosensory deficits and clinical evaluations of hypo-orgasmia (men) and anorgasmia (women). We have previously shown that for humans, other types of sensory input can compensate for deficits caused by loss of PIEZO2 function (11). For example, these individuals use vision to overcome proprioceptive deficits and mechanonociception or thermosensation to mitigate deficits in touch (11). This is also true for human sexual touch (table S1). Nonetheless, the crucial role of PIEZO2 for perineal touch in mice and humans may have therapeutic potential: Topical PIEZO2 inhibitors could provide targeted relief of genital hypersensitivity and pain, whereas agonists of PIEZO2 are candidates for alleviating genital hyposensitivity.

There are a number of limitations to this work. For example, PIEZO2 deficiency is extremely rare, and we were unable to carry out detailed quantitative sensory testing in a larger group of human subjects. Additionally, functional imaging experiments were carried out in anesthetized mice, precluding evaluation of responses during mating. Moreover, although we showed the necessity of gentle touch input for mating, we have not yet demonstrated the sufficiency of this sensory pathway for sexual function in awake behaving animals. We also anticipate that there are likely to be additional specialized roles for mechanosensory neurons in mating that were not revealed in this study.

Even the very gentlest of perineal touches elicits a highly stereotyped startle reaction from mice that is easy to anthropomorphize (movie S1). This PIEZO2-dependent response is quite different from touch to other parts of the body, which typically evokes more modest reactions and does so only at much greater forces. PIEZO2-dependent perineal touch is also a crucial driver of successful mating both for male and female mice. Future studies should help define additional subtypes of sensory neurons needed for sexually dimorphic reactions and how perigenital sensation is organized in the spinal cord and brain to prioritize salience. Ultimately, however, the profound impact of PIEZO2 deficiency that we describe provides a sensory basis at the molecular and cellular level for an aspect of life that throughout history has engaged in human imagination (42) and thought (1).

## Supplementary Material

Supplement

Video 2

Video 1

## Data Availability

All data generated and/or analyzed during the current study are provided in the supplementary materials and/or have been deposited in Dryad.
