# Supplementary material for: PIEZO2 and perineal mechanosensation are essential for sexual function
Source: Science. Author manuscript; Available in PMC 2024 Sep 23. (PMC11418610; doi:10.1126/science.adg0144)
Supplement: Supplement [file NIHMS2021493-supplement-Supplement.pdf]

Science AAAS

Supplementary Materials for

PIEZO2 and perineal mechanosensation are essential for sexual function

Ruby M. Lam *et al*.

Corresponding author: Alexander T. Chesler, alexander.chesler@nih.gov

Science **381**, 897 (2023)
DOI: 10.1126/science.adg0144

The PDF file includes:

Materials and Methods
Figs. S1 to S7
Table S1
References

Other Supplementary Material for this manuscript includes the following:

Movies S1 and S2

Methods

Experimental Model and Subject Details

Clinical evaluation

5 patients with two PIEZO2 loss-of-function alleles were surveyed and evaluated at the National Institutes of Health (NIH) under a research protocol approved by the Institutional Review Boards of National Institute of Neurological Disorders and Stroke (NINDS, protocols 12-N-0095 / 12-N-0077) between April 2015 and May 2023.

Animals

All experiments using animals strictly followed National Institutes of Health (NIH) guidelines and were approved by the National Institute of Neurological Disorders and Stroke (NINDS) or the Scripps Research Institute in compliance with regulatory standards established by the Association for Assessment and Accreditation of Laboratory Animal Care International (AAALAC) Animal Care and Use Committees. Adult male and female mice were used as indicated in the text; for behavioral studies and mating, animals were between 6 and 16 weeks at the start of the experiment; functional imaging was performed on animals weighing between 20 and 35g (10-16 weeks); histology used mice from 5-16 weeks. For all studies, age-matched knockout and wild type littermates were tested at the same age in each cohort. Ai95 (*27*) mice (B6;129S-*Gt(ROSA)26*Sor$^{tm95.1(CAG\text{-}GCaMP6f)Hze}$/J, Jackson Laboratory) were crossed into a *Piezo2*$^{flox/flox}$ background (*21*). Piezo2^{cKO} was induced by intrajugular and intraperitoneal injection of AAV9-CAG-Cre into neonates as described previously (*21*). This approach was extensively quantitated (*21*), results in GCaMP expression in 30-80% of sensory neurons with no apparent selectivity (*34*) and eliminates PIEZO2 function from more than 90% of GCaMP-expressing cells (*21, 34*). Cre-driver lines were used to knockout *Piezo2* in select subsets of neurons using *Hoxb8-Cre (17)*, *Scn10a-Cre* (*31*) and *Pvalb-Cre (27)*. *Scn10a-Cre* was crossed into an Ai9 (B6.Cg-*Gt(ROSA)26Sor*$^{tm9(CAG\text{-}tdTomato)Hze}$ */J*, Jackson Laboratory) background (*43*) for anatomical characterization and to *Piezo2*$^{flox/flox}$ (*12*) or Ai95 to examine function. We also crossed *Piezo2-Cre* (*Piezo2*$^{tm1.1(cre)Apat}$) mice (*26*) with a *Snap25-GFP* (*Snap25*$^{tm1.1Hze}$) Cre-reporter line (*27*) to examine perineal skin projections of cLTMRs.

In vivo epifluorescence calcium imaging of sacral ganglia

Mice were anesthetized with isoflurane and transferred to a custom platform which exposed the genital area for stimulus application. Briefly, the head was loosely secured to the nose cone and hand warmers were used to maintain body temperature. The dorsal aspect of the sacrum was surgically exposed after partial removal of the gluteus medius and stabilized with a spinal clamp (Narishige STS-A).Using a dental drill, the dorsal root ganglia in the pelvis was exposed by removing a portion of the auricular surface along with the posterior articular process of the 6th Vertebra and the first anterior articular process of the sacrum (S1) or posterior articular process (S2); hemostatic dental sponges (Pfizer Gel Foam) were applied as needed to control bleeding. Following surgery, the animal was transferred to the stage of a custom tilting light microscope (Thorlabs Cerna) equipped with a 4X, 0.28 NA air objective (Olympus). GCaMP6f fluorescence images were acquired with a CMOS camera (PCO Panda 4.2) using a standard green fluorescent protein (GFP) filter cube in 40 second epochs at 5 Hz. Mechanical stimuli applied to the animal skin included a series of pressurized air puffs from a Picospritzer (25psi,

for 0.2, 1, 3 and 5 seconds), vibration at (50, 75, 100, 125 and 150 Hz for 3 seconds each), manual gentle brushing with an acrylic brush, skin pinch with forceps (Students) and von Frey filaments stimulation (0.008g, 0.02g, 0.04g, 0.07g, 0.16g, 0.4g, 0.06g, 1.0g, and 1.4g filaments). Regions of interest (ROI) outlining responding cells were drawn in FIJI/ImageJ and relative change of GCaMP6f fluorescence was calculated as percent ΔF/F. Contaminant signal e.g., from out-of-focus tissue and neighboring cells was removed by subtracting the fluorescence of a donut-shaped area surrounding each ROI using a custom MATLAB script (*44*). Overlapping ROIs and rare spontaneously active cells were excluded from the analysis. Imaging episodes were concatenated for display as traces or activity heatmaps. Data shown in figures come from equal numbers of male and female mice.

Background fluorescence noise was calculated for each ROI as the standard deviation of the bottom 25% of all data points as described previously (*44*). A transient rise in fluorescence was considered significant if its peak exceeded 15 times this value. Cells were defined as HTMRs if they responded primarily only to pinch and/or high force von Frey filaments and as LTMRs if they were activated by any or all gentle stimuli. To do this computationally (*34*), cells were classified as air-puff cells if they had significant responses to air-puff that were at least 2 times as strong as their brush response. Vibration cells had significant vibration responses and a ratio of vibration responses to all other responses greater than 3. Brush cells had a significant brush response, a ratio of brush to air-puff response greater than 2 and a ratio of pinch to brush responses greater than 3. Mixed responders had significant air-puff and brush responses with a ratio between 0.5 and 2 and ratios of pinch to low-threshold smaller than 3. Air-puff cells, vibration cells, brush cells and mixed responders all detect low force stimuli and are therefore classified as LTMRs. HTMRs were defined as cells that had a significant response to pinch and had a ratio of high threshold to low threshold peak responses of at least 3. Cells that only responded significantly to von Frey stimulation were also classified as HTMRs.

von Frey thresholds were determined as the smallest force filament eliciting a significant response that was maintained in at least 50% of episodes with increasing filament force.
The area under the curve for a given stimulus and cell was calculated by adding up all ΔF/F data points above baseline from the onset of stimulation to the end of the episode.

Spatial maps of activity were generated by calculating the standard deviation for each pixel over a stimulation episode in FIJI/ImageJ as described previously (*34*). Cell type specific maps (LTMRs vs. HTMRs) were generated by multiplying standard deviation images for the preferred stimulus of each cell with a binary mask outlining the cell. All LTMRs and HTMRs within the field of view were then aggregated by maximum intensity projection. The resulting map distinguishes between LTMRs and HTMRs and provides an estimate of the response magnitude as well as the shape and location of a cell.

von Frey stimulation

Mice were individually habituated on a mesh floor covered by a transparent glass vessel for 1 hour. Nylon monofilaments (Stoelting) were directly applied to the glabrous skin of the hind-paw or to the perineum until the filament bent slightly. Withdrawal, flinch and jump responses within the following 1 second was recorded. This single force touch was repeated 10 times for each filament with ascending forces of (0.008g, 0.02g, 0.04g, 0.07g, 0.16g, 0.4g, 0.06g, 1.0g, and 1.4g) until the animals responded 100% of the time for two filaments in a row. Animals receiving lidocaine treatment were injected intradermally in 5 locations around the genital area (total volume: 125 μl 1% lidocaine).

Female intruder assay and vaginal plug assessment of mating success

All trials probing social and sexual interactions were conducted in home cages without bedding covered by clear plexiglass to allow videography and accurate scoring of behavior. All experiments were conducted during the dark cycle and all animals were sexually naive. Mice were between 8 and 16 weeks of age. In a separate cohorts of 10 mice, blood tests (early light cycle, Ligand Assay and Analysis Core, University of Virginia) confirmed that $Piezo2^{Hoxb8}$ and control males exhibited testosterone and FSH levels that fall within the normal adult range (fig. S7A, B), males had similar gonadal size (mass of dissected testis) relative to total body weight (fig. S7C). We also confirmed that $Piezo2^{Hoxb8}$ and control females exhibited comparable estrus cycle length by examining vaginal lavage on a daily basis at the start of the dark cycle (fig. S7D). Male mice used for female intruder behavior assays were singly housed and adapted to the test chamber for 1 hour. Females were group housed and were evaluated by vaginal lavage and cytology to confirm estrus immediately before their introduction to the test cage. The male was removed after 3 hours; male behavior was scored for the first hour of interaction using BORIS software (*45*). Vaginal copulatory plugs were assessed one hour after the male was removed.

Penile Protrusion Test

Animals, restrained by scruffing, were held in a supine position allowing clear tubing (Tygon S3™ E-3603 F ACFUN007) to be applied around the base of the penis immediately over the external prepuce. Each animal was custom fitted with tubing of optimal inner diameter for gentle prepuce retraction over 5 test trials. Once the correctly sized tubing was determined, 10 tests were performed in succession. The protraction of the internal prepuce as the penis extended into the tube away from the body wall was scored. The test assay was repeated the following day for an additional 10 trials and results pooled.

Histology

Retrograde tracing of sensory innervation used CTB-488, CTB-555, and CTB-647 (ThermoFisher) in 0.2% in PBS. C57Bl/6 Mice were anaesthetized with 2% isoflurane, hair was removed from area of interest and CTB was injected subcutaneously and unilaterally. Paw, glabrous skin (2-4 injections, ~2 µl); perineum (5-10 injections ~4 µl); prepuce / external vagina (2-4 injections ~2 µl); glans (1-2 injections ~2 µl) and opening of vagina (internal vagina,1-2 injections ~2 µl). After 10-14 days, mice were perfused with 4% paraformaldehyde (PFA) in phosphate buffered saline (PBS), the L6-S2 spinal segments and DRGs were dissected and postfixed (4% PFA) overnight at 4°C. The spinal segments and DRGs were transferred to PBS and embedded in a 5% agar/PBS solution. Transverse sections (60 µm for spinal cord and 100 µm for DRGs) were cut on a vibratome. Sections were them mounted on slides and cover slipped with Vectashield Vibrance (Vector laboratories).

To study sensory innervation of the perineum, mice were anesthetized with 2% isoflurane, hair was removed, and the skin cleaned before perfusion with 4% PFA. Skin was dissected, scraped of excess fat and postfixed (4% PFA, overnight). Tissue was washed PBS with 0.3% Triton X-100 (PBST) every hour for 5-8 hours, incubated with primary (1:1000 chicken anti-GFP or rabbit anti-RFP, Abcam) in PBST containing 10% donkey serum and 20% DMSO (DS PBST, room temperature, 3–5 days). Primary antibody was removed with PBST washes (5–8, 1h) and transferred to secondary antibodies (1:200, donkey anti-chicken-FITC or anti-rabbit-Rhodamine-Red-X, Jackson ImmunoResearch) in DS PBST, room temperature, 2

days. Tissue was then washed with PBS every hour for 5 hours and dehydrated in 50%, 75, 100% methanol for 1 hour each, and then left in 100% methanol overnight. Finally, the skin was cleared in BABB (benzyl alcohol; benzyl benzoate, 1:2, Sigma) at room temperature and mounted on slides. Images were acquired using an Olympus BX63L confocal microscope with 10X (air) or 30X (oil) objectives.

For whole-mount ISH, fresh DRGs were dissected from animals and processed as previously described (*34*). Whole unmounted DRGs were dissected and placed into 0.2 ml tubes with 4% paraformaldehyde in phosphate buffered saline (PBS) for 90 mins on ice. Ganglia were washed 3 times in PBS and ISH was performed using Hybridization Chain Reaction (version 3, Molecular Instrument) as previously described (*34*). For RNAscope ISH (Biotechne), fresh frozen sections were collected and processed according to the manufacturer's instructions.

Study design and statistics

No study size calculations or randomization were used in experimental design. Investigators were not blinded to study groups where PIEZO2 mutants displayed obvious phenotypic traits e.g., $Piezo2^{Hoxb8}$, $Piezo2^{Pvalb}$, $Piezo2^{cKO}$ or for human subjects. Investigators were blinded for studies of $Piezo2^{Scn10a}$ mice that do not display a readily observable deficit. When applicable (i.e., for von Frey, pinprick, penile protrusion test, and female intruder assay) scoring was done by two separate investigators and results were pooled. Statistical analysis was performed in Graphpad Prism or Python/Scipy and included two-tailed t-test, parametric one-way ANOVA and Tukey's multiple comparison test for normally distributed data as well as non-parametric Mann-Whitney U test, one-way ANOVA on ranks (Kruskal-Wallis H) followed by Dunn's multiple comparison test for data where normality cannot be assumed. Proportions of binary data were analyzed with a two-tailed Fisher's exact test.

Statistical reporting

Fig. 1A-C. Nonparametric T-tests (Mann-Whitney) were used to compare male and female groups (n=12 in each group); wildtype perineal responses were different P<0.0001 whereas hind-paw responses p=0.4600, and $Piezo2^{Hoxb8}$ perineal touch p >0.9999 were not significantly different. No significant differences between males and females were found for pinprick responses; T-test(parametric); male vs female hind-paws p=0.6893 t=0.4052, df=22; wildtype perineum. p=0.4078 t=0.8439, df=22; $Piezo2^{Hoxb8}$ perineum p>0.9999 t=0.000, df=22 (n=12 in each group).

Male and female data points were pooled to compare von Frey response thresholds and pinprick responses between the 3 groups (Hind-paw, wildtype perineum and $Piezo2^{Hoxb8}$ perineum, n=24 in each group). For von Frey responses statistically significant difference were found between all three groups: one-way ANOVA on ranks (Kruskal-Wallis test) H(df=2,n=72)=62.03 p<0.0001 followed by Dunn's multiple comparison test: hind-paw vs wildtype perineum, p<0.0001; wildtype perineum vs $Piezo2^{Hoxb8}$ perineum p<0.0001; hind-paw vs $Piezo2^{Hoxb8}$ perineum p=0.0006. For pinprick, no statistically significant difference were found between all three groups: parametric one-way ANOVA F(df=2,72)= 1.027 p=0.3635, followed by Tukey's multiple comparison test: hind-paw vs wildtype genitals p= 0.8010; wildtype genitals vs $Piezo2^{Hoxb8}$ genitals p= 0.3311; hind-paw vs $Piezo2^{Hoxb8}$ genitals p=0.7076.

Fig. 2E. Numbers of graded von Frey responsive neurons in control and Piezo2^{cKO} (control n=8, Piezo2^{cKO} n=6) were analyzed by Mann Whitney U test: 0.008g U=5.5 p=0.0087; 0.02g U=0, p=0.0012; 0.04g U=0, p=0.0012; 0.07g U=0, p=0.0012; 0.16g U=0, p=0.0012; 0.4g U=0, p=0.0012; 0.6g U=0, p=0.0012; 1.0g U=2.0 p=0.0027; 1.4g U=1.0 p<0.0018.

Fig. 3A. Quantification of Penile protrusion test. control vs Piezo2^{Hoxb8} Mann-Whitney U (n=18 control,10 Piezo2^{Hoxb8}) p<0.0001; Piezo2^{Scn10a} vs Piezo2^{Pvalb} Mann-Whitney U (n=10 Piezo2^{Scn10a},10 Piezo2^{Pvalb}) p<0.0001.

Fig. 3E. Proportions of females displaying vaginal plugs were analyzed with a two tailed Fisher's exact test. C57Bl/6 vs Piezo2^{Hoxb8} p=0.0031; Piezo2^{Pvalb} vs Piezo2^{Scn10a} p=0.0325.

fig. S3D. Mean area under the curve (AUC) of fluorescence signals was analyzed by a two tailed t-test. LTMRs (paw n=108, genitals n=307): 0.008g t=2.56 p=0.011; 0.02g t=3.43 p=0.00067; 0.04g t=3.29 p=0.0011; 0.07g t=3.66 p=0.000043; 0.16g t=4.67 p<0.00001; 0.4g t=4.13 p<0.00001; 0.6g t=4.34 p=0.000017; 1.0g t=4.02 p=0.000068; 1.4g t=4.66 p<0.00001. HTMRs (paw n=257, genitals n=233): 0.008g t=1.49 p=0.14 n.s.; 0.02g t=2.36 p=0.018; 0.04g t=2.52 p=0.012; 0.07g t=3.33 p=0.00093; 0.16g t=4.67 p<0.00001; 0.4g t=4.89 p<0.00001; 0.6g t=4.19 p=0.000033; 1.0g t=5.65 p<0.00001; 1.4g t=7.46 p<0.00001.

fig. S4F. Numbers of LTMRs and HTMRs per mouse (control n=8, Piezo2^{cKO} n=6) were analyzed by Mann Whitney U test, LTMRs were different U=0 p=0.0012, HTMRs were not significantly different U=23.5 p=0.5.

fig. S7. Piezo2^{Hoxb8} mice have normal sex hormone levels, genital development and estrus cycling. (A, B) Sex-hormone level was determined for 10 age matched control and Piezo2^{Hoxb8} males; a range of ages were used to mirror the ages of mice used for behavioral and functional imaging experiments. FSH and testosterone fall within the normal range for all animals: FSH levels were not significantly different between Control (n=10) and Piezo2^{Hoxb8} males (n=10); unequal variances T-test (Welches) t(df=16.14) = 0.3472, p = 0.7329 ns; testosterone levels were not significantly different between Control (n=10) and Piezo2^{Hoxb8} males (n=10); unequal variances T-test (Welches) t(df=12.43) = 1.193, p = 0.2552 ns. (C) There was no difference in testis-mass relative to mouse body weight between Control (n=4) and Piezo2^{Hoxb8} (n=4); unequal variances T-test (Welches) t(df=3.752) = 1.304 P value=0.2666 ns. (D) Estrus was monitored for groups of 10 control and Piezo2^{Hoxb8} females over a period of 20 days, demonstrating number of estrus cycles was not significantly different between Control (n=10) and Piezo2^{Hoxb8} females (n=10); unpaired T-test t(df=18) = 1.524 p=0.1449 ns.

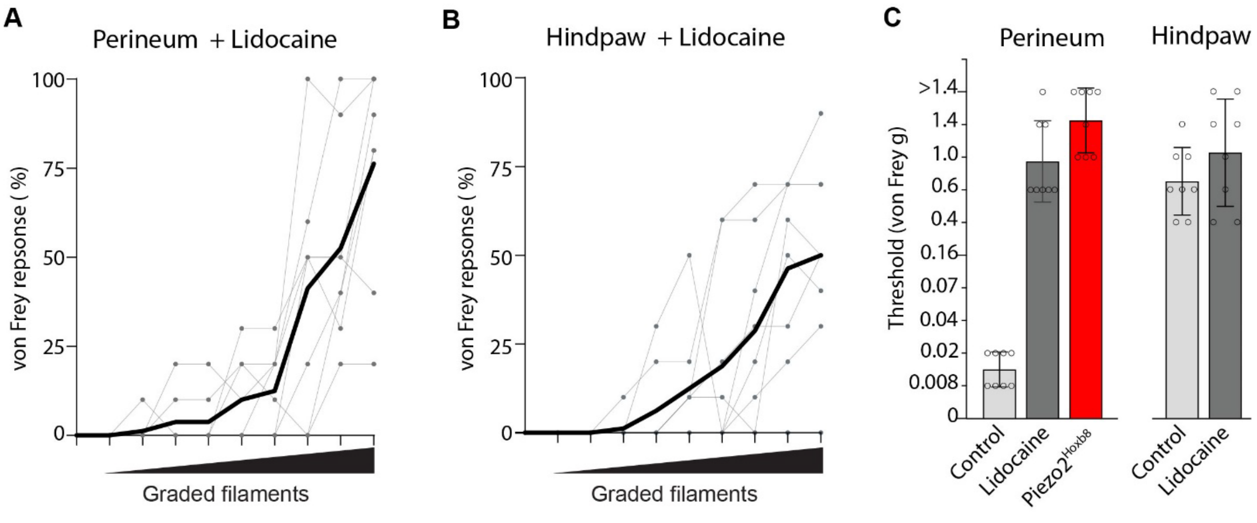

fig. S1. Local anesthetic inhibition of peripheral touch. Reaction of mice to punctate touch of (**A**) the perineum and (**B**) the hindpaw after application of the local anesthetic lidocaine to the test region (n = 8 animals, 4 male, 4 female). Left panels show the responses for individual mice (points and thin lines) and mean (solid lines) to the standard series (Fig. 1) of graded von Frey filaments (each tested 10 times per mouse). (**C**) Quantification of von Frey behavior (threshold defined as ≥ 5/10) and includes data for control and Piezo2^{Hoxb8} mice replotted from Fig. 1 for comparison. Lidocaine treatment increased the threshold for punctate touch detection in the perineum (p= 0.0145) making sensitivity indistinguishable from that of Piezo2^{Hoxb8} mice (p>0.9999 n.s.). The hind-paw was less sensitive than the perineum to this type of touch (Fig. 1) and threshold was not significantly changed by lidocaine treatment (p>0.9999 n.s.). Kruskal-Wallis test H (df=2, n=40) =24.19 p<0.0001 followed by Dunn's multiple comparison test. Male and female data points were pooled for statistical analysis.

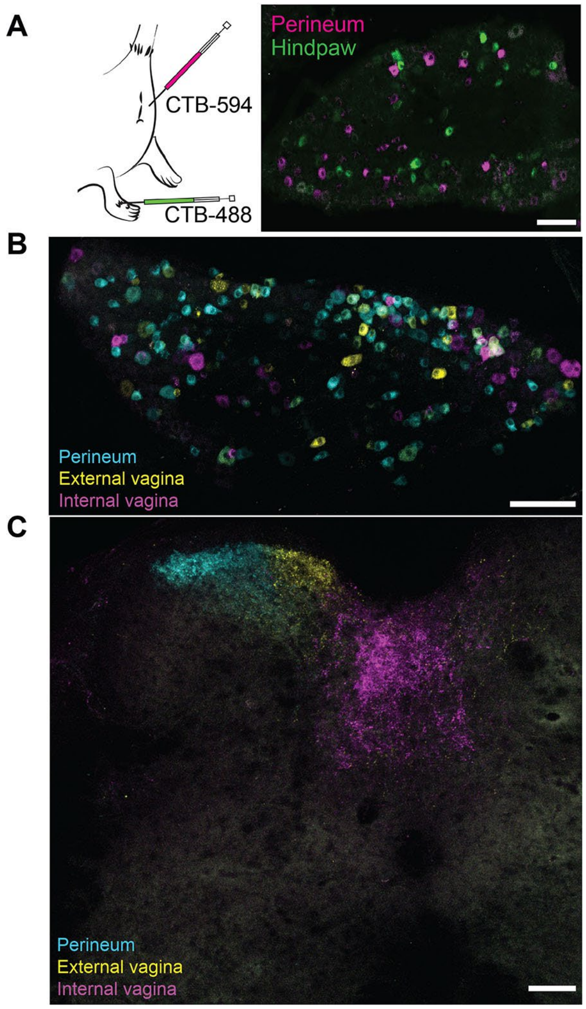

fig. S2. Characterization of sacral DRG neurons innervating the genitals. A) Dual color retrograde CTB labeling from the hind paw (green) and genitals (magenta) of a male mouse illustrating rich innervation of both targets by separate populations of sensory neurons with soma in a sacral ganglion; similar patterns of labeling were observed in both S1 and S2 ganglia (n = 2 male, 2 female). (**B, C**) Triple color retrograde CTB tracing from the perineum (cyan), external vagina / prepuce (yellow) and internal vagina (magenta) showing (**B**) cell bodies of lumbar-sacral sensory neurons in the DRG and (**C**) termini in the dorsal spinal cord (n=2 mice); scale bars = 100 µm.

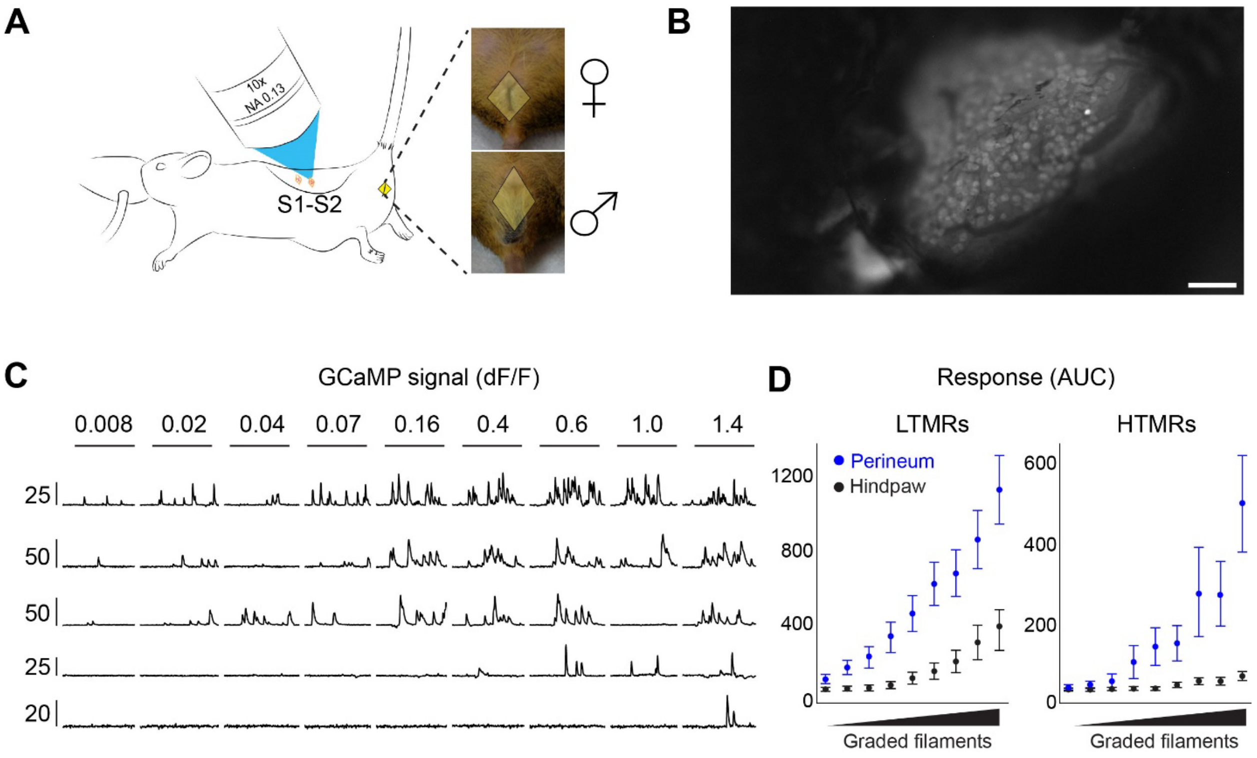

fig. S3. Functional imaging to measure mechanosensory differences between perineal and paw innervating neurons. A) An *in vivo* sacral ganglion Ca-imaging preparation was modified to characterize perineal mechanoreceptors. (**B**) Baseline image of a sacral ganglion prepared for functional imaging; scale bar = 200 µm, see Movie S2 for details of stimulation and response. (**C**) Five representative example GCaMP6f transients from perineal neurons that exhibited differential genital von Frey sensitivity normalized to their maximal response (scale bars, left, ΔF/F, %). (**D**) Quantitation of von Frey stimulation of the perineum and paws for LTMRs (left) and HTMRs (right): the mean area under the curve (AUC) per cell is displayed for each von Frey filament (mean ± 95% confidence interval). Perineal stimulation resulted in significantly larger cell responses for all filaments in LTMRs (two-tailed t-test, $p<0.012$) and for filaments ≥ 0.02g in HTMRs ($p<0.018$).

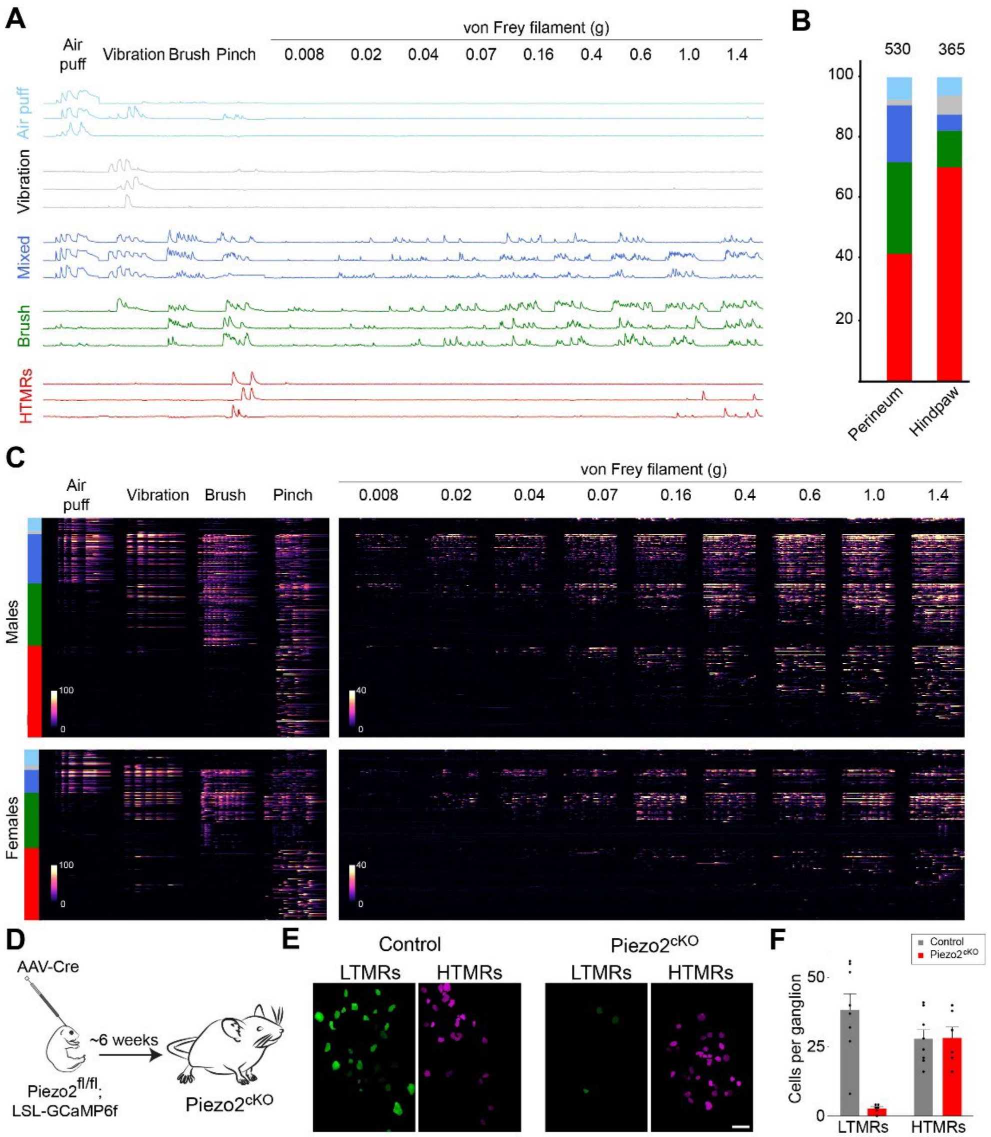

fig. S4. Functional diversity of perineal mechanosensitive neurons and the role of PIEZO2. **A**) Representative GCaMP6f transients from categorized neurons showing 4 classes of LTMRs that respond to one or more types of gentle stimulus and HTMRs that only detect noxious mechanical stimulation (pinch, see Methods). (**B**) Relative abundance of categorized neurons for perineum or hind-paw and total numbers of mechanosensory neurons (perineum, n=8 mice, 4 male, 4 female; hind-paw n=4 mice, 2 male, 2 female). (**C**) Heatmaps representing GCaMP6f responses from perineal mechanosensory neurons to repetitive application of naturalistic stimuli (left panels) and graded von Frey (right panels). Responses from male and female mice (n=4 each) are separated and displayed by functional category (indicated by colored bars); scale: ΔF/F responses. **D**) Schematic of strategy for generating and functionally characterizing perineal *Piezo2* knockout neurons (Piezo2^{cKO}). (**E-F**) LTMRs and HTMRs were defined by their mechanosensory responses (see Methods). (**E**) Example activity maps showing responding LTMRs and HTMRs for control and Piezo2^{cKO}; standard deviation ΔF/F responses, scale bar = 50 µm. (**F**) Quantitation of LTMRs and HTMRs per ganglion (control n=8, Piezo2^{cKO} n=6, same number of males and females; mean ± s.e.m.); LTMRs were significantly reduced by Piezo2^{cKO} (Mann Whitney U test, p=0.0012), HTMRs: no significant differences (Mann Whitney U test, p=0.5 n.s.).

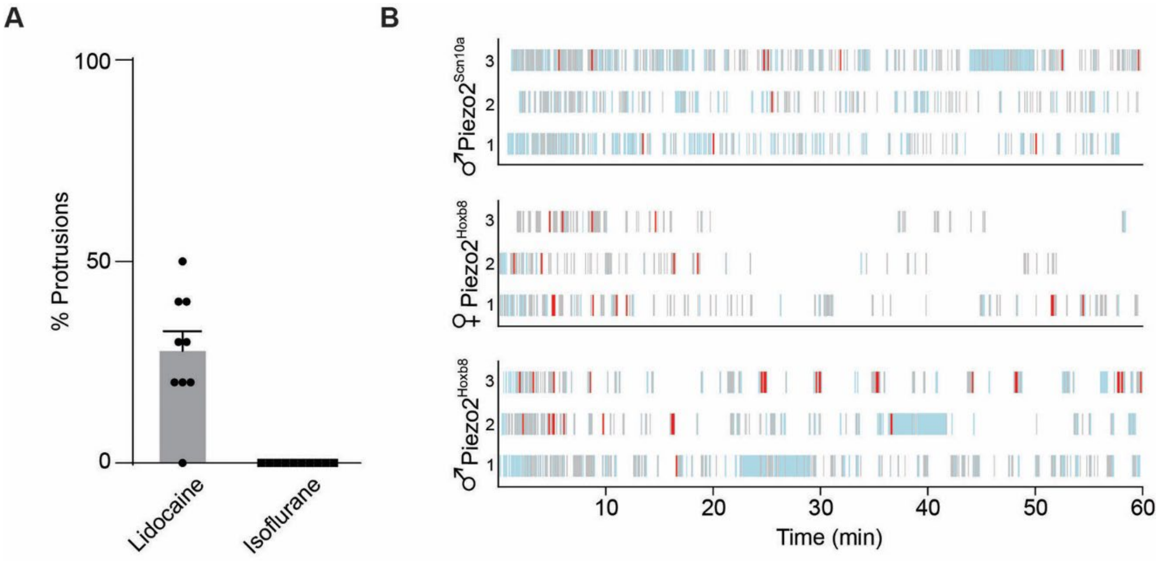

fig. S5. Perineal sensation profoundly affects physiological reflex responses but not sexual motivated behaviors. A) Physiological responses of male mice to perineal stimulation with transparent soft tubing. Penile protrusion into the tube was scored for two sets of ten trials (bars are mean ± s.e.m., individual responses shown as points). Local (lidocaine) and general (2% isoflurane) anesthesia significantly decreased penile protrusion events relative to controls (Mann-Whitney U-test $p<0.0001$, n= 18, Control, see Fig. 4; and n= 9, Lidocaine; n=10, Isoflurane). (**B**) Sexual motivation was assessed using a female intruder assay for additional combinations of mice that exhibited deficits in mating success (plug formation); behaviors scored include social interaction (gray), anogenital investigation (pale blue), and mounting attempts; shown are representative ethogram plots for 3 Piezo2^{Scn10a} males x C57Bl/6 females, 3 Piezo2^{Hoxb8} females x C57Bl/6 males and 3 Piezo2^{Hoxb8} males x C57Bl/6 females for the first hour after introduction of the female in estrus.

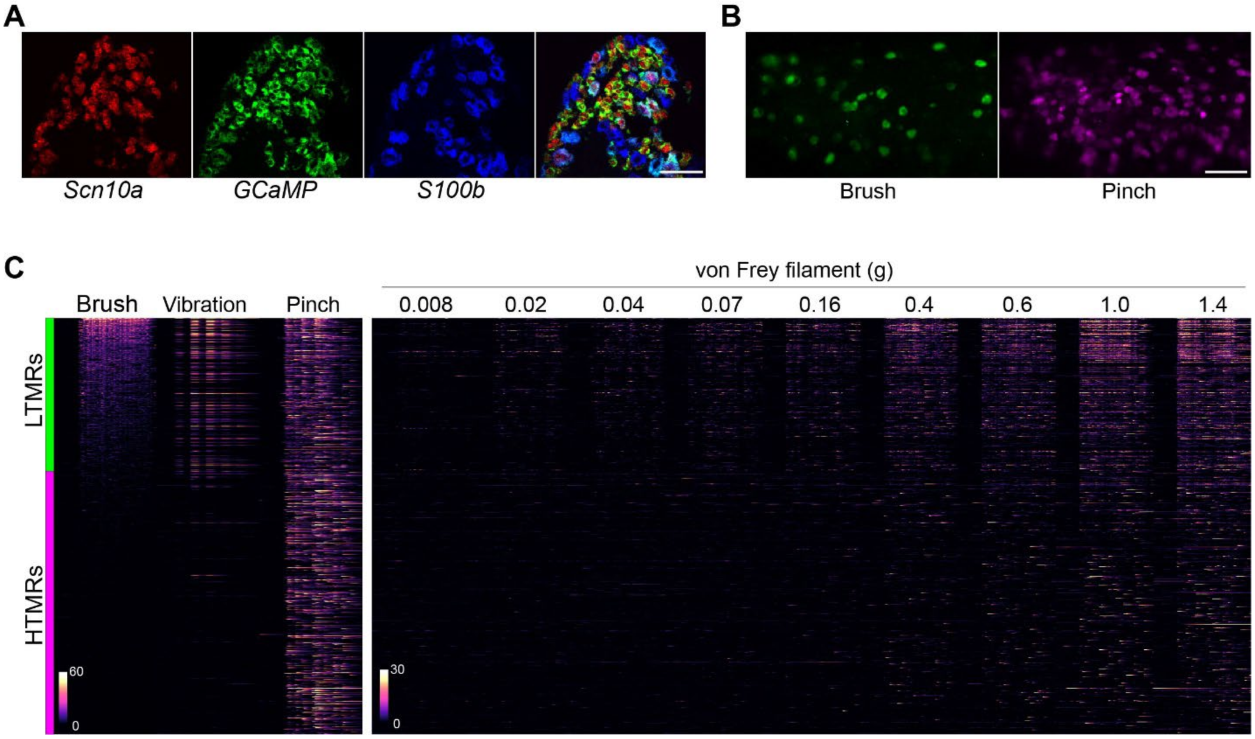

fig. S6. Characterization of genital-innervating neurons in *Scn10a-Cre*::Ai95 mice. A) Example ISH image of sacral ganglion section probed for expression of *Scn10a* (red), *GCaMP* (green-GFP probe) and *S100b* (blue) illustrating the fidelity of recombination (*GCaMP*-expression) in *Scn10a*-positive neurons. Note that there are very few *S100b*-positive, *Scn10a*-negative A-LTMRs that express *GCaMP*; similar results were observed in 5 ganglia. (**B**) Example maximum projection Ca-imaging responses of sacral DRG neurons in *Scn10a-Cre*::Ai95 mice to perineal brush (green) or pinch (magenta). Note the uniform size of brush responding LTMRs and their correspondence with the size of pinch responsive HTMRs, indicating that the vast majority are cLTMRs; scale bar = 50 µm, similar results were observed in 5 mice. (**C**) Heatmaps representing GCaMP6f responses to repetitive application of naturalistic stimuli (left panels) and graded von Frey stimulation (right panels); ΔF/F responses are represented by color coding 0-60%, left panels, 0-30%, right panels. The relative abundance of LTMRs (37%) and HTMRs (63%) is indicated to the left of the heatmaps.

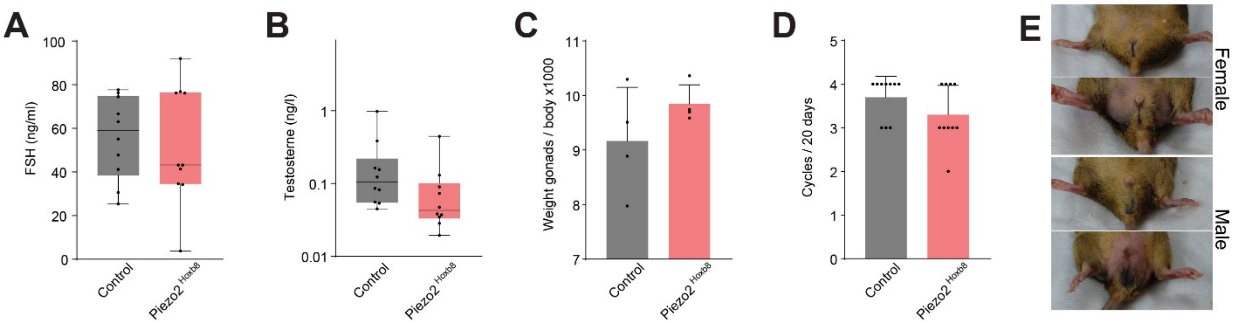

fig. S7. Piezo2^{Hoxb8} mice have normal sex hormone levels, genital development and estrus cycling. A, B) Sex-hormone level was determined for 10 age matched control and Piezo2^{Hoxb8} males; a range of ages were used to mirror the ages of mice used for behavioral and functional imaging experiments. Both (**A**) FSH and (**B**) testosterone fall within the normal range for all animals, similarly, (**C**) there was no difference in testis-mass relative to mouse body weight (n=4, per group). (**D**) Estrus was monitored for groups of 10 control and Piezo2^{Hoxb8} females over a period of 20 days, demonstrating similar cycling times for each group. (**E**) Notably, loss of mechanosensation correlated with genital overgrooming observed in Piezo2^{Hoxb8} mice (9/20 females, top panels; 8/20 males, lower panels) but not littermate controls (0/40). Interestingly, an overgrooming phenotype has been observed in male but not female rats with reduced genital sensory input (*46*).

table S1

A. Relevant genotypic and phenotypic details of human subjects

Subject	Male-1	Male-2	Male-3	Female-1	Female-2
Age	20	47	53	35	40
Allele 1	c.7730 C>A<br>Ser2577 ***Stop***	c.2004delG<br>Gly668 ***Stop***	c.6496G>T<br>Glu2166 ***Stop***	c.3241C>T<br>Arg1051 ***Stop***	c.3241C>T<br>Arg1051 ***Stop***
Allele 2	IVS47+1 G>A<br>(***Splice junction***)	c.2004delG<br>Gly668 ***Stop***	c.6496G>T<br>Glu2166 ***Stop***	c.3241C>T<br>Arg1051 ***Stop***	c.3241C>T<br>Arg1051 ***Stop***
Major symptoms	Profound absence of proprioception, vibratory sense and discriminatory touch perception specifically on glabrous skin and deficiency of allodynia without loss of deep pressure, temperature or other pain sensation (*11, 21*).				
Other notes	No reported delayed puberty			No reported delayed puberty or amenorrhea	

B. Responses to genital sensitivity and sexual response related questions

	PIEZO2-LOFs N = 5		Controls N = 10	
	Yes	No	Yes	No
Are you sexually active?	5	0	10	0
Does physical stimulation alone produce arousal?	5	0	9	1
Are erotic thoughts or videos sufficient to produce arousal without touch?	5[a]	0	10	0
Can you perceive external genital sensation?	3[b]	2	10	0
Have you experienced an orgasm?	3	2	9	1
Is physical stimulation and/or sex satisfying?	4	1	10	0
Males	N = 3		N = 5	
Can you distinguish between a squeeze of the gland vs. shaft?	3	0	5	0
Females	N = 2		N = 5	
Is lubrication normal?	0	2	5	0

[a] One male reported that erotic thoughts/videos are "sufficient to produce erection, but not a full erection".

[b] Anecdotes: "If my genitals are being touched by a woman in a subtle way and I can't see it with my eyes, I hardly have any physical of [sic] sexual sensation"; "If the touch is very subtle, I don't feel it."; "Yes, but I need additional stimulation around my genitals, more friction, extra pressure."

Movie S1. Example behavioral responses of wildtype (C57Bl/6, left) and Piezo2^{HoxB8} (right) male mice to 0.04g von Frey filament and pinprick (as indicated) applied to the perineum. Videos were synchronized and slowed down four-times so that application of stimulus and responses of the strains can be compared.

Movie S2. Example of sacral ganglion functional imaging showing application of naturalistic and von Frey stimuli to the perineum and synchronized real time GCaMP6f fluorescence changes in a control male animal.

References and Notes

1. C. Darwin, *The Descent of Man and Selection in Relation to Sex* (Murray, 1871), vols. 1 and 2.
2. J. J. Dinsmore, Courtship Behavior of the greater bird of paradise. *Auk* **87**, 305–321 (1970).
3. S. J. Gould, The origin and function of ‘bizarre’ structures: Antler size and skull size in the ‘Irish elk,’ *Megaloceros giganteus*. *Evolution* **28**, 191–220 (1974).
4. M. A. Changizi, Q. Zhang, S. Shimojo, Bare skin, blood and the evolution of primate colour vision. *Biol. Lett.* **2**, 217–221 (2006).
5. B. D. Charlton, M. S. Martin-Wintle, M. A. Owen, H. Zhang, R. R. Swaisgood, Vocal behaviour predicts mating success in giant pandas. *R. Soc. Open Sci.* **5**, 181323 (2018).
6. V. S. Mandiyan, J. K. Coats, N. M. Shah, Deficits in sexual and aggressive behaviors in Cnga2 mutant mice. *Nat. Neurosci.* **8**, 1660–1662 (2005).
7. L. Stowers, T. E. Holy, M. Meister, C. Dulac, G. Koentges, Loss of sex discrimination and male-male aggression in mice deficient for TRP2. *Science* **295**, 1493–1500 (2002).
8. C. C. Voigt, O. Behr, B. Caspers, O. von Helversen, M. Knörnschild, F. Mayer, M. Nagy, Songs, scents, and senses: Sexual selection in the greater sac-winged bat, *Saccopteryx bilineata*. *J. Mammal.* **89**, 1401–1410 (2008).
9. C. A. Kell, K. von Kriegstein, A. Rösler, A. Kleinschmidt, H. Laufs, The sensory cortical representation of the human penis: Revisiting somatotopy in the male homunculus. *J. Neurosci.* **25**, 5984–5987 (2005).
10. B. Coste, J. Mathur, M. Schmidt, T. J. Earley, S. Ranade, M. J. Petrus, A. E. Dubin, A. Patapoutian, Piezo1 and Piezo2 are essential components of distinct mechanically activated cation channels. *Science* **330**, 55–60 (2010).
11. A. T. Chesler, M. Szczot, D. Bharucha-Goebel, M. Čeko, S. Donkervoort, C. Laubacher, L. H. Hayes, K. Alter, C. Zampieri, C. Stanley, A. M. Innes, J. K. Mah, C. M. Grosmann, N. Bradley, D. Nguyen, A. R. Foley, C. E. Le Pichon, C. G. Bönnemann, The role of PIEZO2 in human mechanosensation. *N. Engl. J. Med.* **375**, 1355–1364 (2016).
12. S. S. Ranade, S.-H. Woo, A. E. Dubin, R. A. Moshourab, C. Wetzel, M. Petrus, J. Mathur, V. Bégay, B. Coste, J. Mainquist, A. J. Wilson, A. G. Francisco, K. Reddy, Z. Qiu, J. N. Wood, G. R. Lewin, A. Patapoutian, Piezo2 is the major transducer of mechanical forces for touch sensation in mice. *Nature* **516**, 121–125 (2014).
13. Y. García-Mesa, L. Cárcaba, C. Coronado, R. Cobo, J. Martín-Cruces, J. García-Piqueras, J. Feito, O. García-Suárez, J. A. Vega, Glans clitoris innervation: PIEZO2 and sexual mechanosensitivity. *J. Anat.* **238**, 446–454 (2021).
14. Y. García-Mesa, J. García-Piqueras, R. Cobo, J. Martín-Cruces, I. Suazo, O. García-Suárez, J. Feito, J. A. Vega, Sensory innervation of the human male prepuce: Meissner’s corpuscles predominate. *J. Anat.* **239**, 892–902 (2021).
15. I. Abdus-Saboor, N. T. Fried, M. Lay, J. Burdge, K. Swanson, R. Fischer, J. Jones, P. Dong, W. Cai, X. Guo, Y.-X. Tao, J. Bethea, M. Ma, X. Dong, L. Ding, W. Luo, Development

of a mouse pain scale using sub-second behavioral mapping and statistical modeling. *Cell Rep.* **28**, 1623–1634.e4 (2019).

16. T. Hua, B. Chen, D. Lu, K. Sakurai, S. Zhao, B.-X. Han, J. Kim, L. Yin, Y. Chen, J. Lu, F. Wang, General anesthetics activate a potent central pain-suppression circuit in the amygdala. *Nat. Neurosci.* **23**, 854–868 (2020).

17. S. E. Murthy, M. C. Loud, I. Daou, K. L. Marshall, F. Schwaller, J. Kühnemund, A. G. Francisco, W. T. Keenan, A. E. Dubin, G. R. Lewin, A. Patapoutian, The mechanosensitive ion channel Piezo2 mediates sensitivity to mechanical pain in mice. *Sci. Transl. Med.* **10**, eaat9897 (2018).

18. V. E. Abraira, D. D. Ginty, The sensory neurons of touch. *Neuron* **79**, 618–639 (2013).

19. K. Nonomura, S.-H. Woo, R. B. Chang, A. Gillich, Z. Qiu, A. G. Francisco, S. S. Ranade, S. D. Liberles, A. Patapoutian, Piezo2 senses airway stretch and mediates lung inflation-induced apnoea. *Nature* **541**, 176–181 (2017).

20. S.-H. Woo, V. Lukacs, J. C. de Nooij, D. Zaytseva, C. R. Criddle, A. Francisco, T. M. Jessell, K. A. Wilkinson, A. Patapoutian, Piezo2 is the principal mechanotransduction channel for proprioception. *Nat. Neurosci.* **18**, 1756–1762 (2015).

21. M. Szczot, J. Liljencrantz, N. Ghitani, A. Barik, R. Lam, J. H. Thompson, D. Bharucha-Goebel, D. Saade, A. Necaise, S. Donkervoort, A. R. Foley, T. Gordon, L. Case, M. C. Bushnell, C. G. Bönnemann, A. T. Chesler, PIEZO2 mediates injury-induced tactile pain in mice and humans. *Sci. Transl. Med.* **10**, eaat9892 (2018).

22. M. L. Sorrells, J. L. Snyder, M. D. Reiss, C. Eden, M. F. Milos, N. Wilcox, R. S. Van Howe, Fine-touch pressure thresholds in the adult penis. *BJU Int.* **99**, 864–869 (2007).

23. C. B. Bleustein, H. Eckholdt, J. C. Arezzo, A. Melman, Quantitative somatosensory testing of the penis: Optimizing the clinical neurological examination. *J. Urol.* **169**, 2266–2269 (2003).

24. L. Li, M. Rutlin, V. E. Abraira, C. Cassidy, L. Kus, S. Gong, M. P. Jankowski, W. Luo, N. Heintz, H. R. Koerber, C. J. Woodbury, D. D. Ginty, The functional organization of cutaneous low-threshold mechanosensory neurons. *Cell* **147**, 1615–1627 (2011).

25. V. E. Abraira, E. D. Kuehn, A. M. Chirila, M. W. Springel, A. A. Toliver, A. L. Zimmerman, L. L. Orefice, K. A. Boyle, L. Bai, B. J. Song, K. A. Bashista, T. G. O'Neill, J. Zhuo, C. Tsan, J. Hoynoski, M. Rutlin, L. Kus, V. Niederkofler, M. Watanabe, S. M. Dymecki, S. B. Nelson, N. Heintz, D. I. Hughes, D. D. Ginty, The cellular and synaptic architecture of the mechanosensory dorsal horn. *Cell* **168**, 295–310.e19 (2017).

26. S. H. Woo, S. Ranade, A. D. Weyer, A. E. Dubin, Y. Baba, Z. Qiu, M. Petrus, T. Miyamoto, K. Reddy, E. A. Lumpkin, C. L. Stucky, A. Patapoutian, Piezo2 is required for Merkel-cell mechanotransduction. *Nature* **509**, 622–626 (2014).

27. L. Madisen, A. R. Garner, D. Shimaoka, A. S. Chuong, N. C. Klapoetke, L. Li, A. van der Bourg, Y. Niino, L. Egolf, C. Monetti, H. Gu, M. Mills, A. Cheng, B. Tasic, T. N. Nguyen, S. M. Sunkin, A. Benucci, A. Nagy, A. Miyawaki, F. Helmchen, R. M. Empson, T. Knöpfel, E. S. Boyden, R. C. Reid, M. Carandini, H. Zeng, Transgenic mice for

intersectional targeting of neural sensors and effectors with high specificity and performance. *Neuron* **85**, 942–958 (2015).

28. K. L. Marshall, D. Saade, N. Ghitani, A. M. Coombs, M. Szczot, J. Keller, T. Ogata, I. Daou, L. T. Stowers, C. G. Bönnemann, A. T. Chesler, A. Patapoutian, PIEZO2 in sensory neurons and urothelial cells coordinates urination. *Nature* **588**, 290–295 (2020).
29. F. A. Beach, Sexual attractivity, proceptivity, and receptivity in female mammals. *Horm. Behav.* **7**, 105–138 (1976).
30. B. D. Sachs, Sexual reflexes of spinal male house mice. *Physiol. Behav.* **24**, 489–492 (1980).
31. N. Agarwal, S. Offermanns, R. Kuner, Conditional gene deletion in primary nociceptive neurons of trigeminal ganglia and dorsal root ganglia. *Genesis* **38**, 122–129 (2004).
32. N. Sharma, K. Flaherty, K. Lezgiyeva, D. E. Wagner, A. M. Klein, D. D. Ginty, The emergence of transcriptional identity in somatosensory neurons. *Nature* **577**, 392–398 (2020).
33. M. Q. Nguyen, C. E. Le Pichon, N. Ryba, Stereotyped transcriptomic transformation of somatosensory neurons in response to injury. *eLife* **8**, e49679 (2019).
34. L. J. von Buchholtz, N. Ghitani, R. M. Lam, J. A. Licholai, A. T. Chesler, N. J. P. Ryba, Decoding cellular mechanisms for mechanosensory discrimination. *Neuron* **109**, 285–298.e5 (2021).
35. C. J. Burnett, S. C. Funderburk, J. Navarrete, A. Sabol, J. Liang-Guallpa, T. M. Desrochers, M. J. Krashes, Need-based prioritization of behavior. *eLife* **8**, e44527 (2019).
36. S. X. Zhang, A. Lutas, S. Yang, A. Diaz, H. Fluhr, G. Nagel, S. Gao, M. L. Andermann, Hypothalamic dopamine neurons motivate mating through persistent cAMP signalling. *Nature* **597**, 245–249 (2021).
37. X. Zhou, A. Li, X. Mi, Y. Li, Z. Ding, M. An, Y. Chen, W. Li, X. Tao, X. Chen, Y. Li, Hyperexcited limbic neurons represent sexual satiety and reduce mating motivation. *Science* **379**, 820–825 (2023).
38. R. D. Johnson, Z. Halata, Topography and ultrastructure of sensory nerve endings in the glans penis of the rat. *J. Comp. Neurol.* **312**, 299–310 (1991).
39. D. Ohmori, Über die entwicklung der innervation der genitalapparate als peripheren aufnahmeapparat der genitalen reflexe. *Anat. Embryol.* **70**, 347–410 (1924).
40. D. Huzard, M. Martin, F. Maingret, J. Chemin, F. Jeanneteau, P.-F. Mery, P. Fossat, E. Bourinet, A. François, The impact of C-tactile low-threshold mechanoreceptors on affective touch and social interactions in mice. *Sci. Adv.* **8**, eabo7566 (2022).
41. H. Olausson, Y. Lamarre, H. Backlund, C. Morin, B. G. Wallin, G. Starck, S. Ekholm, I. Strigo, K. Worsley, Å. B. Vallbo, M. C. Bushnell, Unmyelinated tactile afferents signal touch and project to insular cortex. *Nat. Neurosci.* **5**, 900–904 (2002).
42. M. Vatsyayana, *The Kama Sutra of Vatsyayana*, R. F. Burton, B. Indrajit, S. P. Bhide, Transl. (1883).
43. L. Madisen, T. A. Zwingman, S. M. Sunkin, S. W. Oh, H. A. Zariwala, H. Gu, L. L. Ng, R. D. Palmiter, M. J. Hawrylycz, A. R. Jones, E. S. Lein, H. Zeng, A robust and high-

throughput Cre reporting and characterization system for the whole mouse brain. *Nat. Neurosci.* **13**, 133–140 (2010).

44. N. Ghitani, A. Barik, M. Szczot, J. H. Thompson, C. Li, C. E. Le Pichon, M. J. Krashes, A. T. Chesler, Specialized mechanosensory nociceptors mediating rapid responses to hair pull. *Neuron* **95**, 944–954.e4 (2017).

45. O. Friard, M. Gamba, BORIS: A free, versatile open-source event-logging software for video/audio coding and live observations. *Methods Ecol. Evol.* **7**, 1325–1330 (2016).

46. C. L. Moore, Sex differences in self-grooming of rats: Effects of gonadal hormones and context. *Physiol. Behav.* **36**, 451–455 (1986).
